# Roles of Nitric Oxide and Prostaglandins in the Sustained Antihypertensive Effects of* Acanthospermum hispidum* DC. on Ovariectomized Rats with Renovascular Hypertension

**DOI:** 10.1155/2017/2492483

**Published:** 2017-09-20

**Authors:** Rhanany Alan Calloi Palozi, Maysa Isernhagen Schaedler, Cleide Adriane Signor Tirloni, Aniely Oliveira Silva, Francislaine Aparecida dos Reis Lívero, Roosevelt Isaias Carvalho Souza, Ariany Carvalho dos Santos, Thiago Bruno Lima Prando, Lauro Mera de Souza, Arquimedes Gasparotto Junior

**Affiliations:** ^1^Faculdade de Ciências da Saúde, Universidade Federal da Grande Dourados, Rodovia Dourados-Itahum, Km 12, P.O. Box 533, 79.804.970 Dourados, MS, Brazil; ^2^Laboratório de Pesquisa Pré-Clínica de Produtos Naturais, Universidade Paranaense, Praça Mascarenhas de Moraes 4282, P.O. Box 224, 87.502-210 Umuarama, PR, Brazil; ^3^Instituto de Pesquisa Pelé Pequeno Príncipe, Faculdades Pequeno Príncipe, Av. Iguaçu 333, 80.230.020 Curitiba, PR, Brazil

## Abstract

Although* Acanthospermum hispidum* is used in Brazilian folk medicine as an antihypertensive, no study evaluated its effects on a renovascular hypertension and ovariectomy model. So, this study investigated the mechanisms involved in the antihypertensive effects of an ethanol-soluble fraction obtained from* A. hispidum* (ESAH) using two-kidney-one-clip hypertension in ovariectomized rats (2K1C plus OVT). ESAH was orally administered at doses of 30, 100, and 300 mg/kg, daily, for 28 days, after 5 weeks of surgery. Enalapril (15 mg/kg) and hydrochlorothiazide (25 mg/kg) were used as standard drugs. Diuretic activity was evaluated on days 1, 7, 14, 21, and 28. Systolic, diastolic, and mean blood pressure and heart rate were recorded. Serum creatinine, urea, thiobarbituric acid reactive substances, nitrosamine, nitrite, aldosterone, vasopressin levels, and ACE activity were measured. The vascular reactivity and the role of nitric oxide (NO) and prostaglandins (PG) in the vasodilator response of ESAH on the mesenteric vascular bed (MVB) were also investigated. ESAH treatment induced an important saluretic and antihypertensive response, therefore recovering vascular reactivity in 2K1C plus OVT-rats. This effect was associated with a reduction of oxidative and nitrosative stress with a possible increase in the NO bioavailability. Additionally, a NO and PG-dependent vasodilator effect was observed on the MEV.

## 1. Introduction

In recent years, evidence has shown that there are significant differences between the genesis and the development of cardiovascular diseases between men and women [1]. In general, women are more affected by cardiovascular diseases, especially hypertension, after a pronounced drop in estrogen levels, a fact that usually occurs after menopause. It is now known that one of the mechanisms by which blood pressure may be elevated in aging postmenopausal women is the activation of the renin-angiotensin system (RAS). Postmenopausal women exhibit an increase in plasma renin activity, suggesting activation of the RAS similarly to what occurs during renovascular hypertension [2]. Approximately 50% of menopausal women have hypertension, a condition that associated with other estrogen-related risk factors (such as obesity and dyslipidemia) significantly contributes to acute cardiovascular events [3]. In contrast, although this condition is quite common, preclinical studies specifically designed for this type of population are still very restricted [4].

Recently, we have shown that the ethanol-soluble fraction obtained from* Acanthospermum hispidum* DC. (Asteraceae) (ESAH), an important medicinal species widely used in Brazil, has a significant acute hypotensive effect on normotensive male Wistar rats [5]. In addition, we have also shown that ESAH, rich in phenolic compounds, such as derivatives of caffeic acid and glycosylated flavonoids (quercetin glucoside and galactoside), does not produce any toxic effects after acute treatment [5]. So, despite the widespread use of* A. hispidum* as an antihypertensive by the Brazilian population [6], its prolonged diuretic and antihypertensive effects have not yet been scientifically evaluated.

Thus, in this work, the prolonged diuretic and antihypertensive effects of* A. hispidum* on ovariectomized rats with renovascular hypertension were investigated to simulate a broad part of the woman population aged over 50 years. In addition, the molecular mechanisms involved in* A. hispidum* antihypertensive response using isolated mesenteric vascular bed (MVB) were also evaluated.

## 2. Materials and Methods

### 2.1. Drugs

The following drugs, salts, and solutions were used: xylazine and ketamine hydrochloride (from Syntec, São Paulo, SP, Brazil), heparin (from Hipolabor, Belo Horizonte, MG, Brazil), and acetylcholine chloride, phenylephrine, indomethacin, N*ω*-nitro-L-arginine methyl ester (L-NAME), sodium nitroprusside, NaCl, KCl, NaHCO_3_, MgSO_4_, CaCl_2_, KH_2_PO_4_, dextrose, and ethylenediaminetetraacetic acid (EDTA) (all purchased from Sigma-Aldrich, Saint Louis, MO, USA).

### 2.2. Phytochemical Study

#### 2.2.1. Plant Material and Preparation of the Purified Aqueous Extract

Aerial parts of* Acanthospermum hispidum* were collected from the botanical garden of the Federal University of Grande Dourados (UFGD, Dourados, Brazil) at 458 m above sea level (S 22°11′42.7′′ and W 54°56′10.2′′), in October 2015. A voucher specimen was authenticated by Dra. Maria do Carmo Vieira (DDMS number 5219) and deposited at the UFGD plant facility.

*A. hispidum* aqueous extract was obtained by infusion in a similar manner to that popularly used in Brazil [6] and prepared according to Tirloni et al. [5]. For this,* A. hispidum* leaves were air-dried in an oven at 40°C for 7 days and then the dried plant was cut and ground into powder form using mechanical milling. The extract was obtained by infusing 1 liter of boiling water for each 60 grams of dried and pulverized plant. Extraction was carried out until room temperature was reached (~5 h). The infusion was treated with 3 volumes of EtOH, which gave rise to a precipitate and an ethanol-soluble fraction (ESAH). ESAH was filtered, ethanol was totally removed by evaporation, and the resulting fraction was lyophilized (yield of 8.05%). All preparations were kept in freezer until further analyses.

#### 2.2.2. Sample Analysis (Liquid Chromatography-Mass Spectrometry (LC-MS))

Chromatography was performed in an ultra-performance liquid chromatography (UPLC™ Waters), using a reversed phase column HSS T3 C18 column, with 100 × 2.1 mm and 1.7 *μ*m of particle size (Waters), with constant temperature of 60°C. The solvents were ultra-pure water (Milli-Q, Millipore) and acetonitrile (JT Baker) containing 0.1% (v/v) of formic acid 96% (Tedia), and the gradient was performed increasing the acetonitrile from 0 to 10%, in 6 min, then to 80% in 14 min, at flow rate of 0.4 ml/min. The solvent returned to the initial condition in 15 min and the system was reequilibrated for 2 min. The sample was prepared at 2 mg/mL in MeOH-H_2_O (1 : 1, v/v), with injections of 5 *μ*L.

The detection was provided by a high-resolution mass spectrometer, LTQ Orbitrap XL (Thermo Scientific). The ions were detected in the negative and positive modes. The ion source was held at 350°C and the desolvation was aided by nitrogen stream, at 40 arbitrary units in the sheath gas and 10 a.u. in the auxiliary gas. Negative ions provided best results for the sample, with the energies at 3.5 kV in the source, −46 V in the capillary, and −200 V in the tube lens. Fragmentations were performed by higher-energy collisional dissociation, with normalized collision energy of 25–35. For LC-MS analysis, the MS resolution was set at 15,000 FWHM mass accuracy which was obtained by external calibration, routinely performed.

### 2.3. Pharmacological Studies

#### 2.3.1. Animals

Twelve-week-old female Wistar rats weighing 200–300 g were randomized and housed in plastic cages, with environmental enrichment, at 22 ± 2°C under a 12/12 h light dark cycle, 55 ± 10% humidity conditions, with* ad libitum *access to food and water. All procedures were approved by the ethical committee on animal use of the Federal University of Grande Dourados (UFGD) (number 45/2016), and experiments were performed in accordance with international standards and ethical guidelines on animal welfare.

#### 2.3.2. Ovariectomy and Induction of Renovascular Hypertension

Rats were anesthetized with ketamine (100 mg/kg) and xylazine (20 mg/kg) by intraperitoneal route. After ovariectomy (OVT), renovascular hypertension was induced using the Goldblatt model (two kidneys, one clip; 2K1C) as described by Umar et al. [7]. The left renal artery was exposed by retroperitoneal incision and dissected. A silver clip (lumen of 0.22 mm) was placed around the artery for partial occlusion. In the Sham-operated group (placebo surgery), the artery was not clipped and ovaries were not removed. After surgery, animals received saline for rehydration (2 ml/animal, subcutaneously, single administration), anti-inflammatory (indomethacin, 2 mg/kg, by oral route, every 12 hours, during 3 days), and antibiotic (enrofloxacin, 10 mg/kg, subcutaneously, single dose). Systolic blood pressure (SBP) was weekly measured (for 4 weeks) using the tail-cuff method. Only hypertensive rats (SBP above 140 mm Hg) were used in experiments.

#### 2.3.3. Experimental Groups

Five weeks after surgery, animals were randomized and divided into 7 groups (*n* = 6-7) for hemodynamic and renal function studies. Rats were treated once a day (by gavage), during 28 days, with vehicle (2K1C plus OVT = positive control group), hydrochlorothiazide (HCTZ, 25 mg/kg, standard diuretic drug), enalapril (ENAL, 15 mg/kg, standard antihypertensive drug), or ESAH (30, 100, or 300 mg/kg). The Sham-operated group received vehicle under the same conditions.

#### 2.3.4. Diuretic Activity

The diuretic effects of ESAH were accessed according to methods previously described by Kau et al. [8] with some modifications [9]. Weekly (on days 1, 7, 14, 21, and 28), rats were fasted overnight (6 h) with free access to water. Animals received an oral load of isotonic saline (0.9% NaCl, 5 ml/100 g) to impose a controlled water and salt balance before treatments. Then, rats were immediately placed in metabolic cages. Urine samples were collected in a graduated cylinder and the volume was recorded 8 hours after treatment (expressed as ml per 100 g body weight). During the experiment, rats were food deprived. pH and density were determined on fresh urine samples using digital pH meter (Q400MT; Quimis Instruments, Brazil) and a handheld refractometer (NO107; Nova Instruments, Brazil), respectively. Urinary sodium (Na^+^), potassium (K^+^), chloride (Cl^−^), and calcium (Ca^++^) levels were quantified in automated analyzer (Roche® Cobas Integra 400 plus). Excretion load (El) of Na^+^, K^+^, Cl^−^, and Ca^++^ was obtained according to the equation El = *U*_*x*_ × *V*, where *U*_*x*_ is the concentration of electrolytes (mEq/L) and *V* is the urinary flow (mL/min). Results were expressed as *μ*Eq/min/100 g.

#### 2.3.5. Blood Pressure and Heart Rate Evaluation

At the end of the trial period (day 28), rats were anesthetized with ketamine (100 mg/kg) and xylazine (20 mg/kg) by intramuscular route. Immediately, a* bolus* injection of heparin (50 IU) was subcutaneously applied. The left carotid artery was cannulated and connected to a pressure transducer coupled to a PowerLab® recording system, and an application software (Chart, v 4.1; all from ADI Instruments, Castle Hill, Australia), recording systolic blood pressure (SBP) and diastolic blood pressure (DBP), mean blood pressure (MBP), and heart rate (HR). After 15 minutes of stabilization, changes in blood pressure and HR were recorded for 5 min.

#### 2.3.6. Mesenteric Vascular Reactivity

After systemic blood pressure and HR measurements, and before euthanasia, the mesenteric vascular bed (MVB) was isolated and prepared for perfusion as previously described [10]. The isolated MVBs were then placed in a water-jacketed organ bath maintained at 37°C and perfused with PSS (composition in mM: NaCl 119; KCl 4.7; CaCl_2_ 2.4; MgSO_4_ 1.2; NaHCO_3_ 25.0; KH_2_PO_4_ 1.2; dextrose 11.1; and EDTA 0.03) gassed with 95% O_2_/5% CO_2_ at constant flow rate of 4 ml/min through a peristaltic pump. Changes in the perfusion pressure (mm Hg) were measured by a pressure transducer connected to an acquisition system (PowerLab) and its application software (Chart, v 7.1; both from ADI Instruments, Castle Hill, Australia). After setup in the perfusion apparatus, preparation was allowed to equilibrate for 30 to 45 min, and its viability was checked by a* bolus* injection of KCl (120 mmol). After a 30-minute stabilization period, a phenylephrine (PHE; 3, 10, and 30 nmol) dose-response administration was performed by injecting increasing doses into the perfusion system. After a 60-minute equilibration period, MVBs were continuously perfused with PSS containing 3 *μ*M PHE, which was enough to induce a sustained increased perfusion pressure. Under these conditions, vasodilatory effects of acetylcholine (ACh; 3, 10, and 30 pmol) or sodium nitroprusside (SNP; 3, 10, and 30 pmol) were measured. A 15-minute equilibration period was allowed between each drug. All drugs were given into the perfusate as bolus injection of 100 *μ*l.

#### 2.3.7. Serum Biochemical Parameters

At the end of experiments, all animals were killed by suprapharmacological isoflurane dose and blood samples were collected. Serum was obtained by centrifugation (1,000 ×g for 10 min). Serum Na^+^, K^+^, urea, and creatinine were determined in automated biochemical analyzer (Roche Cobas Integra 400 plus). Serum nitrotyrosine (NT), aldosterone, and vasopressin levels were measured by enzyme-linked immunosorbent assay kit according to manufacturer's specifications (BD Biosciences, CA, USA). Thiobarbituric acid (TBARS) levels were measured using TBARS assay kits (Cayman Chemical, Ann Arbor, Michigan, USA) according to manufacturer's instruction. Plasma nitrite concentration was determined by enzymatically reducing nitrate according to technique described by Schmidt et al. [11]. Serum angiotensin converting enzyme (ACE) activity was determined by indirect fluorimetry according to methods described by Santos et al. [12].

### 2.4. Investigation of the Molecular Mechanisms Involved in the Vasodilatory Response of ESAH

#### 2.4.1. Evaluation of the Effects of ESAH on the Mesenteric Vascular Bed

Preparations (MVBs) with functional endothelium from normotensive and hypertensive (2K1C plus OVT) female rats, without any previous treatment, were continuously perfused with PSS containing PHE (3 *μ*M). After stabilization of the increased perfusion pressure, preparations received* bolus* injections containing 0.0003, 0.001, 0.003, and 0.01 mg of ESAH, and the reduction in the perfusion pressure was evaluated. Each next dose was administered only after the return of the perfusion pressure to the same level recorded before injection, with minimal interval of 3 min between doses.

#### 2.4.2. Investigation of the Mechanisms Involved in the Vascular Effects of ESAH

In these experiments, after recording the first dose-response curve to ESAH (0.001, 0.003, and 0.01 mg), the MVBs were left to equilibrate for an additional period of 30–45 min under perfusion with PSS. Then, different preparations were perfused with PSS containing 3 *μ*M phenylephrine plus indomethacin (1 *μ*M; a nonselective cyclooxygenase inhibitor) and L-NAME (100 *μ*M; a nonselective NO synthase inhibitor) alone or combined. After 15 min under perfusion with one of the previously mentioned solutions, ESAH (0.001, 0.003, and 0.01 mg) was injected again into the perfusion system. The ability of ESAH to reduce the perfusion pressure was compared with results obtained in control preparations, perfused with vehicle only.

### 2.5. Statistical Analyses

Data were analyzed for homogeneity of variance and normal distribution. Differences between means were determined using one-way analysis of variance (ANOVA) followed by Dunnett's* post hoc *test. The level of significance was set at 95% (*p* < 0.05), and results are expressed as mean ± standard error of the mean (SEM). Graphs were drawn and statistical analysis was carried out using GraphPad® Prism software version 5.0 for Mac OS X (GraphPad Software, San Diego, CA, USA).

## 3. Results

### 3.1. Phytochemical Characterization

In a previous work [6], the composition of an extract of* Acanthospermum hispidum* was partially characterized, with identification of the family of monocaffeoylquinic acids (CQAs) and dicaffeoylquinic acids (diCQAs), observed as multiple isomers in the LC-MS analysis. However, these isomers have characteristic fragmentation profile that can be used for their identification [13, 14]. To confirm the isomer identity, an extract of* Ilex paraguariensis*, containing mono- and dicaffeoylquinic acids, previously characterized [13], was used as a reference sample.

**Figure 1:**
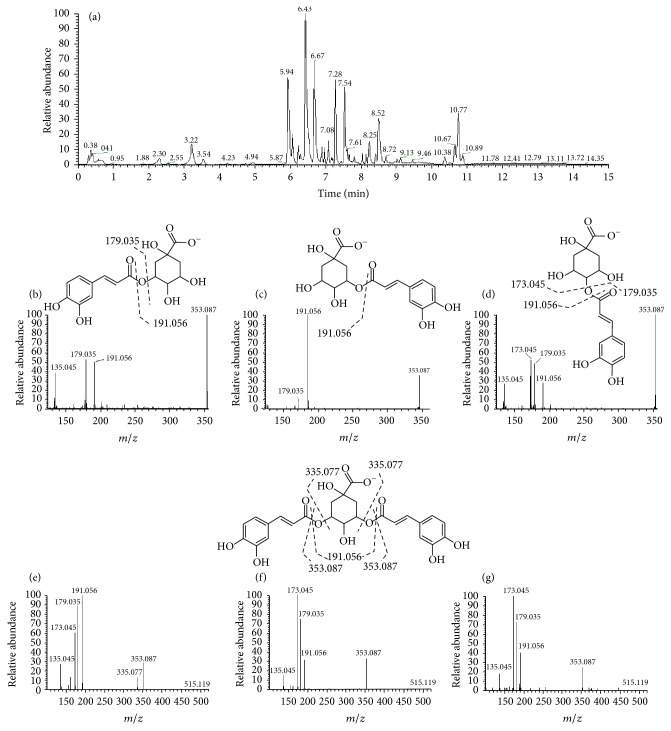
Phytochemical analysis of ESAH obtained from* A. hispidum* DC. (a) Base peak chromatogram obtained by UPLC detected by HR-MS in the negative ionization and the fragmentation profile of monocaffeoylquinic acids, tentatively identified as* neo*-chlorogenic acid (5-CQA) (b), chlorogenic acid (3-CQA) (c), and* crypto*-chlorogenic acid (4-CQA) (d). The main breakdown site for dicaffeoylquinic acids is depicted in the 3,5 dicaffeoylquinic acid (3,5-di-CQA) and the fragmentation profile observed for peak 6.43 min (e), 6.67 min (f), and 7.07 min (g).

The detailed chemical composition of ESAH obtained from* A. hispidum* is shown in Table 1. The negative ionization was better for the acidic compounds present in the extract, and the monocaffeoylquinic acids appeared at *m*/*z* 353.087. The carbons from the ring of quinic acid were numerated according to the chlorogenic acid structure, considered as 3-monocaffeoylquinic acid (3-CQA). Thus, based on the fragmentation profile (at collision energy of 25) and retention time, compared to* I. paraguariensis* extract, the isomers could be predicted as* neo*-chlorogenic acid (5-CQA at 2.30 min), with intense fragments at *m*/*z* 191.056, 179.035, and 135.045 but absent of *m*/*z* 173.45. The chlorogenic acid (3-CQA) appeared at 3.22 min, with a main fragment at *m*/*z* 191.056 and the* crypto*-chlorogenic acid (4-CQA at 3.54 min) produced fragments at *m*/*z* 191.056, 179.035, 173.045, and 135.045, detaching the most abundant fragment-ion at *m*/*z* 173.045 (Figure 1). These results are in accordance with the pioneer work of Carini et al. [15]. The peaks at 5.94 and 6.05 min were observed at *m*/*z* 463.088, with similar fragments at *m*/*z* 300.027, 271.024, 255.029, and 243.029, consistent with quercetin-hexosides, being consistent with quercetin galactoside and quercetin glucoside [16].

The main peaks on chromatogram were identified as dicaffeoylquinic acids (di-CQAs) at *m*/*z* 515.119, and similar to the CQAs, they differ in their fragmentation profile obtained with collision energy at 35. The peak at 6.43 min was consistent with 3,4-di-CQA, with fragments at *m*/*z* 353.087 due to the loss of a caffeoyl residue, and the fragment at *m*/*z* 335.076 (353.087, H_2_O) was observed at a relative intensity in only this isomer. The other fragments were *m*/*z* 191.056 (quinic acid, base peak), *m*/*z* 179.035 (caffeic acid), and *m*/*z* 173.045 (quinic acid, H_2_O). The peaks at 6.67 and 7.07 min presented a quite similar fragmentation profile, containing almost all fragments observed for the peak at 6.43 min, except by the absence of ion at *m*/*z* 335.076. The chromatographic profile suggests the peak at 6.67 min as 3,5-di-CQA and the peak at 7.07 min as 4,5-di-CQA. However, other isomers may be eluted in very close retention time as previously observed [14], hindering the accurate isomer identification. Other peaks (7.28, 7.54, and 8.52 min) showed the main ion at *m*/*z* 337.238, which could be result of in-source fragmentation and remains unidentified and the peaks at 10.38, 10.67, 10.77, and 10.89 min presented multiple ions avoiding their identification.

### 3.2. Prolonged Treatment with ESAH Induced Significant Saluretic Effect on Sham-Operated and 2K1C Plus OVT-Rats

The values obtained for urinary volume and renal excretion of electrolytes are shown in Tables 2, 3, 4, 5, and 6. The mean urinary volumes of Sham-operated rats collected for 8 hours on days 1, 7, 14, 21, and 28 were, respectively, 5.64 ± 0.70, 9.16 ± 1.09, 10.36 ± 1.32, 8.98 ± 0.93, and 7.84 ± 0.55 ml/100 g/8 h. Similarly, 2K1C plus OVT-animals produced an estimated urinary volume of 4.99 ± 0.27, 5.56 ± 0.21, 7.11 ± 0.62, 7.16 ± 0.42, and 5.92 ± 0.32 ml/100 g/8 h on days 1, 7, 14, 21, and 28, respectively. None of the groups treated with ENAL or ESAH (30, 100, and 300 mg/kg) had any changes in urinary volume when compared to Sham-operated or 2K1C plus OVT-rats. As expected, animals that received HCTZ had a significant increase in urinary volume only on the 1st day of treatment.

On the 1st day of treatment, all animals treated with ESAH (30, 100, or 300 mg/kg) or HCTZ presented a significant increase in Na^+^ and Cl^−^ elimination, with saluretic index higher than 2.0 (Table 2). On the other hand, only animals treated with ESAH (30, 100, and 300 mg/kg) maintained this effect on the 7th day of treatment (Table 3). On the 14th, 21st, and 28th days of treatment, only animals receiving ESAH at the dose of 300 mg/kg had significant urinary Na^+^ and Cl^−^ levels (Tables 4, 5, and 6). In contrast, animals treated with HCTZ returned to significant Na^+^ levels only on the 28th day of treatment (Table 6). Groups receiving ENAL did not show any significant increase in urinary Na^+^, K^+^, Cl^−^, or Ca^++^ when compared with positive controls or Sham-operated animals. Similarly, urinary pH and density or serum Na^+^, K^+^, urea, and creatinine values were not altered by any of the treatments (Tables 2, 3, 4, 5, 6, and 7).

### 3.3. ESAH Administration Reduces SBP, DBP, MBP, and HR in 2K1C Plus OVT-Rats

**Figure 2:**
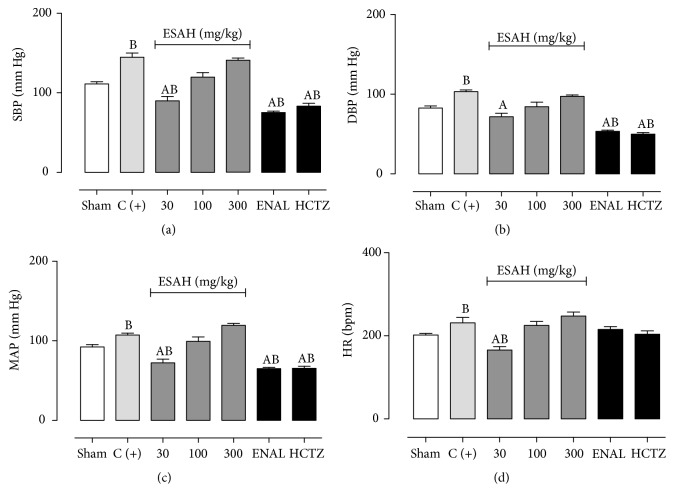
Prolonged administration of ESAH obtained from* A. hispidum* reduces SBP (a), DBP (b), MAP (c), and HR (d) in 2K1C plus OVT-rats. Vehicle, ESAH (30, 100, and 300 mg/kg), ENAL (15 mg/kg), or HCTZ (25 mg/kg) was given orally for 28 days. The letter “C+” indicates the effect measured after administration of vehicle only. The results show the mean ± SEM (*n* = 6-7). Statistical analyses were performed by means of one-way analysis of variance (ANOVA) followed by Dunnett's test. ^A^*p* < 0.05 when compared to 2K1C plus OVT-rats (C+). ^B^*p* < 0.05 when compared to Sham-operated group. ENAL: enalapril; DBP: diastolic blood pressure; HCTZ: hydrochlorothiazide; HR: heart rate; MAP: mean arterial pressure; SBP: systolic blood pressure; Sham: placebo surgery.

Figures 2(a), 2(b), 2(c), and 2(d) show the systemic blood pressure and HR values obtained after prolonged treatment with ESAH, ENAL, or HCTZ. All animals in the positive control group presented significant increase in SBP (144 ± 5.3 versus 111 ± 3.7 mm Hg), DBP (103 ± 2.8 versus 82 ± 2.9 mm Hg), MBP (110 ± 3.8 versus 92 ± 2.8 mm Hg), and HR values (251 ± 12 versus 202 ± 4.6 bpm) when compared to Sham-operated rats. Prolonged administration of ESAH at doses of 30 mg/kg was able to reduce SBP, DBP, and MBP to 90 ± 5.5, 71 ± 4.4, and 77 ± 5.5 mm Hg, respectively, while HR levels were reduced to 165 ± 7.7 bpm. In addition, ESAH at doses of 100 and 300 mg was not able to change the SBP, DBP, MBP, or HR of normotensive or 2K1C plus OVT-rats. However, as expected, ENAL and HCTZ were able to significantly reduce SBP, DBP, and MBP levels in positive controls or Sham-operated animals. Values obtained with ENAL or HCTZ administration on the arterial pressure levels were not statistically different from those observed after treatment with ESAH (30 mg/kg).

### 3.4. Prolonged Treatment with ESAH Restores Vascular Reactivity in MVBs from 2K1C OVT-Rats

**Figure 3:**
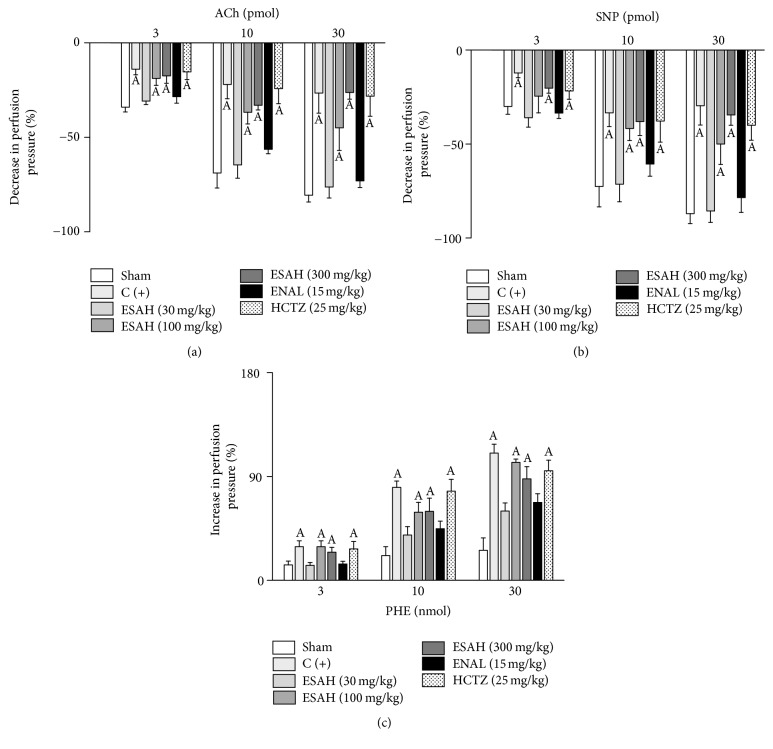
Prolonged treatment with ESAH restores vascular reactivity in MVBs from 2K1C plus OVT-rats. Effects of ACh (3, 10, and 30 pmol; (a)), SNP (3, 10, and 30 pmol; (b)), or PHE (3, 10, and 30 nmol; (c)) on the perfusion pressure of the MVBs from normotensive (Sham) or 2K1C plus OVT-rats in the presence or absence of prolonged treatment with ESAH (30, 100, 300 mg/kg), ENAL (15 mg/kg), or HCTZ (25 mg/kg). Values in panel are expressed as mean ± SEM of 5 experiments. A indicates *p* < 0.05 compared with the perfusion pressure in Sham-operated rats. All experiments were performed in endothelium-intact preparations. ACh: acetylcholine; ENAL: enalapril; HCTZ: hydrochlorothiazide; SNP: sodium nitroprusside; OVT: ovariectomy; PHE: phenylephrine.

In MVBs from 2K1C plus OVT-rats, the administration of ACh or SNP was able to induce a vasodilatory response approximately 35% lower than that in Sham-operated rats (Figures 3(a) and 3(b)). Similarly, the vasoconstrictor response of PHE was significantly higher in the 2K1C plus OVT-group when compared to Sham-operated animals (Figure 3(c)). On the other hand, in animals that received ESAH (30 mg/kg) or ENAL (15 mg/kg), the effects of ACh, SNP, or PHE were not different from those observed in Sham-operated animals. Treatments with HCTZ (25 mg/kg) or ESAH at doses of 100 and 300 mg/kg were not able to reverse the changes in vascular reactivity observed in 2K1C plus OVT-animals (Figures 3(a)–3(c)).

### 3.5. ESAH Reduces Oxidative and Nitrosative Stress without Affecting Aldosterone, Vasopressin Levels, or Serum ACE Activity

**Figure 4:**
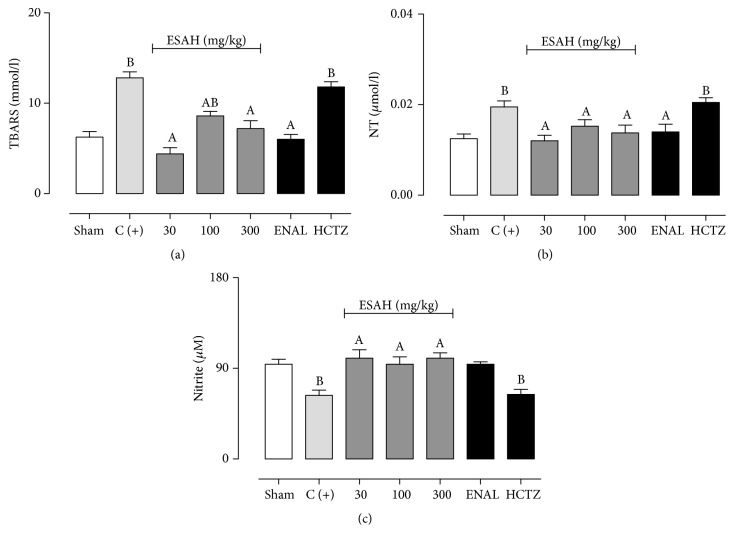
ESAH treatment reduces TBARS (a) and NT (b) levels and increases NO bioavailability (c). The serum samples were obtained after 28 days of treatment with vehicle, ESAH (30, 100, and 300 mg/kg), ENAL (15 mg/kg), or HCTZ (25 mg/kg). The results show the mean ± SEM (*n* = 6-7). Statistical analyses were performed by means of one-way analysis of variance (ANOVA) followed by Dunnett's test.. ^A^*p* < 0.05 when compared to 2K1C plus OVT-rats (C+). ^B^*p* < 0.05 when compared to Sham-operated group. ENAL: enalapril; HCTZ: hydrochlorothiazide; NT: nitrosamine; Sham: placebo surgery; TBARS: thiobarbituric acid reactive substances.

The effects of ESAH, ENAL, and HCTZ on TBARS, NT, and nitrite levels are shown in Figures 4(a), 4(b), and 4(c). Ovariectomy associated with renovascular hypertension increases TBARS and NT levels in ~104 and 60%, respectively. Treatment with ESAH (30, 100, and 300 mg/kg) or ENAL reduced TBARS and NT levels to values close to those found in Sham-operated animals.

At baseline, nitrite concentration was significantly lower in positive control animals when compared with Sham-operated ones (63 ± 5.3 versus 94 ± 5.0 *μ*M). On the other hand, treatment with ESAH (at all doses) or ENAL increased the nitrite levels by ~50%.

**Figure 5:**
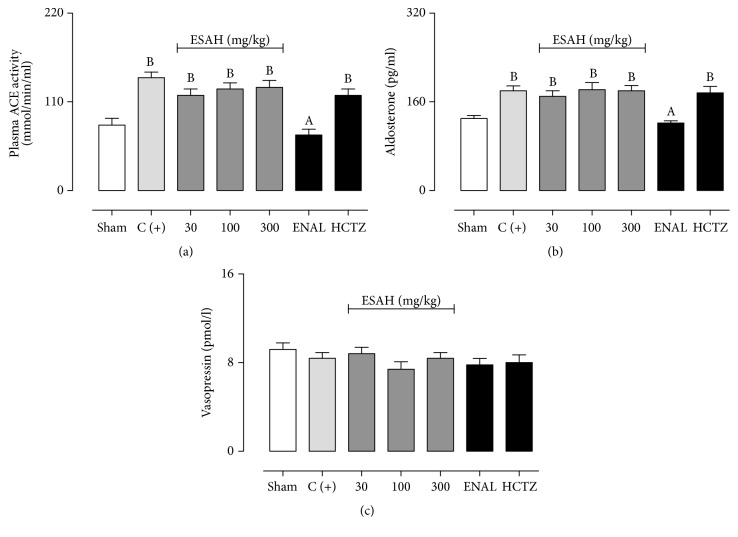
Prolonged treatment with ESAH does not affect plasma ACE activity (a) or serum levels of aldosterone (b) and vasopressin (c). The serum samples were obtained after 28 days of treatment with vehicle, ESAH (30, 100, and 300 mg/kg), ENAL (15 mg/kg), or HCTZ (25 mg/kg). The results show the mean ± SEM (*n* = 6-7). Statistical analyses were performed by means of one-way analysis of variance (ANOVA) followed by Dunnett's test. ^A^*p* < 0.05 when compared to 2K1C plus OVT-rats (C+). ^B^*p* < 0.05 when compared to Sham-operated group. ACE: angiotensin converting enzyme; ENAL: enalapril; HCTZ: hydrochlorothiazide; Sham: placebo surgery.

On the other hand, only ENAL was able to reduce the aldosterone levels and serum ACE activity (Figures 4(a)–4(c)), without affecting serum vasopressin. Treatment with ESAH (30, 100, and 300 mg/kg) or HCTZ did not significantly change the aldosterone and vasopressin concentrations or serum ACE activity (Figures 5(a)–5(c)).

### 3.6. ESAH Induce Vasodilatory Response in MVBs from Normotensive and 2K1C Plus OVT-Rats in Dependence on Endothelial NO and Prostaglandins

**Figure 6:**
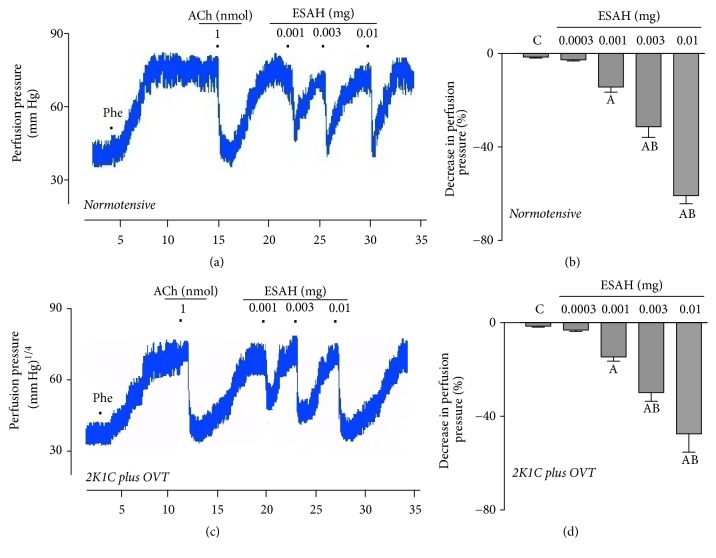
ESAH induces vasodilatory response in MVBs from normotensive and 2K1C plus OVT-rats. Effects of ESAH (0.001, 0.003, and 0.01 mg) on the perfusion pressure of the MVBs from normotensive (b) and 2K1C plus OVT-rats (d), perfused with physiological saline solution containing 3-*μ*M phenylephrine (PHE). Trace recording of the MVBs pressure showing the effects of the administration of acetylcholine and ESAH in normotensive (a) and 2K1C plus OVT-rats (c). Values in panel are expressed as mean ± SEM of 5 experiments. A indicates *p* < 0.05 compared with the perfusion pressure recorded before ESAH. B indicates *p* < 0.05 compared with the previous dose. All experiments were performed in endothelium-intact preparations.

The continuous perfusion of the MVBs (from normotensive or 2K1C plus OVT-rats) with phenylephrine resulted in a sustained increase in the vascular perfusion pressure, which was significantly reduced by ESAH (0.001, 0.003, and 0.01 mg) (Figures 6(b) and 6(d)). The characterization of a typical experiment shown in Figure 6(a) (normotensive) or Figure 6(c) (2K1C plus OVT-rats) reveals that when the highest ESAH dose was used (0.01 mg), the vasodilatory effects of ESAH had response intensity similar to that of acetylcholine.

**Figure 7:**
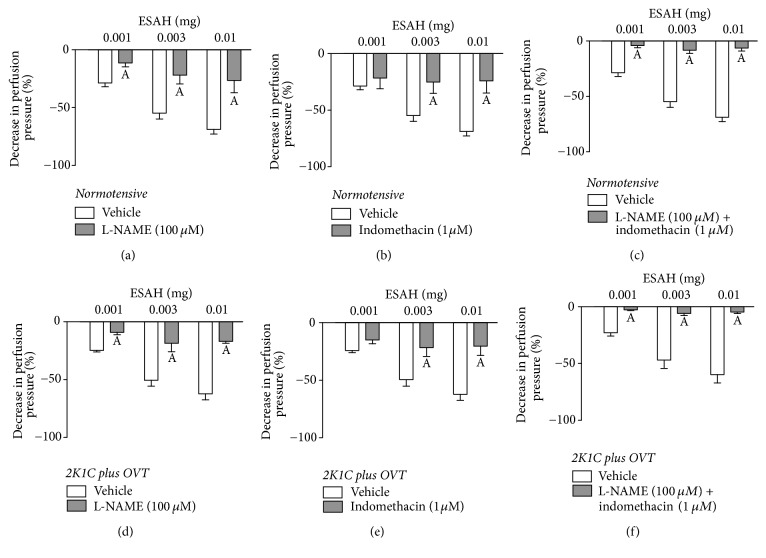
Role of NO and prostaglandins on the vasorelaxant effect of ESAH in MVBs from normotensive and hypertensive rats (2K1C plus OVT). Effects of ESAH (0.001, 0.003, and 0.01 mg) on endothelium-intact MVBs continuously perfused with L-NAME ((a) and (d)), indomethacin ((b) and (e)), or coadministration of L-NAME and indomethacin ((c) and (f)). The results show the mean ± SEM of 5 preparations per group. A indicates *p* < 0.05 compared with the effects of ESAH on the respective vehicle group.

Previous infusion with L-NAME significantly reduced part of the effect on the MVBs obtained with ESAH. The peak effect of 0.001, 0.003, and 0.01 mg of ESAH was decreased from 28 ± 4%, 54 ± 6%, and 68 ± 7% to 11 ± 3%, 22 ± 9%, and 26 ± 11% in normotensive and from 24 ± 3%, 50 ± 7%, and 62 ± 9% to 9 ± 2%, 18 ± 7%, and 16 ± 6% in 2K1C plus OVT-rats, respectively (Figures 7(a) and 7(d)). In a similar way, the vasodilatory effect of ESAH at doses of 0.001, 0.003, and 0.01 mg was reduced to 20 ± 7%, 25 ± 8%, and 27 ± 9% in normotensive and to 14 ± 3%, 21 ± 6%, and 20 ± 7% in 2K1C plus OVT-rats, in preparations perfused with indomethacin (Figures 7(b) and 7(e)). Interestingly, simultaneous treatment (coadministration) with L-NAME and indomethacin (Figures 7(c) and 7(f)) vanished the vasorelaxation effect induced by all ESAH doses in MVBs from normotensive or 2K1C plus OVT-rats.

## 4. Discussion

In the present study, it was demonstrated that 28-day ESAH treatment was effective in inducing an important saluretic and antihypertensive response in 2K1C plus OVT-rats. This favorable effect induced by ESAH is associated with a parallel reduction of oxidative and nitrosative stress biomarkers, in addition to a possible increase in NO bioavailability. Additionally, an important NO and prostaglandin-dependent vasodilator effect was observed in mesenteric vascular bed, a finding that indicates a potential mechanism for the cardiovascular effects of ESAH.

In the first stage of our study, we decided to use ENAL and HCTZ as standard cardioprotective drugs. The choice of HCTZ was based on the need to compare the diuretic and natriuretic effects of ESAH with a first-line diuretic drug in the treatment of hypertension [17]. On the other hand, as the renovascular hypertension model used in our study (2K1C) induces a sustained activation of the renin-angiotensin system (RAS), we chose another classic drug (ENAL) capable of effectively blocking activation and preventing the installation of renovascular hypertension. In addition, data indicated that ACE inhibitors, including ENAL, would exert cardioprotective effects regardless of the hypotensive action [18]. Current findings have indicated that ACE inhibitors may increase NO bioavailability and reduce oxidative and nitrosative stress parameters during hypertension. These effects provide an additional cardioprotective response to the sustained antihypertensive effect [19]. Therefore, considering our findings, ENAL was presented as a standard drug for comparison with the cardioprotective potential of ESAH.

Another point that deserves attention is the initiation of treatments with ESAH, ENAL, and HCTZ only 4 weeks after the surgical procedure. This option stems from the need to submit animals to a previous period of sustained activation of RAS and estrogen deprivation, a limiting step in oxidative/nitrosative imbalance and in the establishment of hypertension. In fact, all 2K1C plus OVT-rats had a high degree of oxidative/nitrosative stress, accompanied by increased vascular reactivity to PHE and a reduced vasodilatory response to ACh and SNP, showing a significant change in endothelial and vascular smooth muscle function. In addition, we observed a significant increase in ACE activity and aldosterone levels, which may have contributed directly to the onset of hypertension. Treatment with ESAH significantly reduced TBARS and NT levels and increased nitrite concentration (an indirect marker of NO bioavailability), showing important antioxidant and antinitrosant properties. In addition, ESAH treatment restored vascular reactivity to PHE, ACh, and SNP, demonstrating its significant vasoprotective effects in 2K1C plus OVT-rats. In fact, this response may play a central role in the cardiorenal effects of ESAH, since, unlike ENAL, ESAH did not affect ACE activity and aldosterone levels.

When we look closely at the renal effects of ESAH, we will see that while HCTZ showed an expected diuretic response, with an increase in water and electrolyte elimination after first administration, followed by a reduction of the diuretic effect by compensatory mechanisms, ESAH did not affect the urine volume eliminated. On the other hand, an important saluretic effect was evident, especially on the elimination of Na^+^ and Cl^−^ throughout the experimental period. In fact, at the lowest doses (30 and 100 mg/kg), the saluretic effect was evident only on the 1st and 7th days of treatment, while renal Na^+^ and Cl^−^ elimination continued significantly only at the highest dose tested (300 mg/kg). If we look at the antihypertensive effect of ESAH, we will see that the most significant reduction in blood pressure levels also occurred at the lowest dose used. This fact may explain in part the reduction of the saluretic response observed after the 7th day of treatment, since an eventual reduction of renal hydrostatic pressure, due to the sustained reduction of blood pressure, can reduce glomerular filtration rate and renal salt and water elimination. This effect was also evident with HCTZ, a classic antihypertensive drug that, despite inducing significant diuretic effect in the first dose, showed an important reduction in its efficacy after prolonged treatment.

It is known that cardiorenal regulation may involve several endogenous mediators, including prostaglandins, NO, and the endothelium-derived hyperpolarizing factor, synergistically acting in a complex hemodynamic and neurohumoral interaction [20]. In our study, it has been shown that the cardiorenal effects induced by ESAH appear to be influenced by an expressive antioxidant activity, which could influence the increase in the bioavailability of NO and consequently the vascular reactivity. Although suggestive, there is still some doubt whether the effect is due to direct release of NO by secondary metabolites present in ESAH or a consequence of the potent antioxidant activity. The fact is that substances that reduce reactive oxygen species may increase the bioavailability of NO and prostaglandins and consequently induce systemic vasodilator responses [21].

In order to prove this hypothesis, we evaluated the ability of ESAH to reduce perfusion pressure in the MVBs from normotensive and 2K1C plus OVT-rats in the absence and presence of L-NAME and indomethacin, two drugs capable of inhibiting NO and prostaglandin synthase, respectively. The putative blockade of NO and prostaglandin synthesis prevented the reduction of perfusion pressure induced by ESAH in the MVBs, suggesting that the release of NO and prostaglandins could be involved in this effect. A data that reinforces our findings was described in a recent study [5] where it shows that an extract with a similar phytochemical profile was able to induce a NO-dependent acute hypotensive response in normotensive rats, evidencing the importance of NO in the cardiovascular effects of ESAH. Although we believe that the cardiorenal effects presented in this study result from a complex interaction among different secondary metabolites present in the ESAH, the fact that there may be one or more agents involved cannot be ruled out. Biomonitoring studies can help answer these questions and, if possible, identify the agent that stands out in this process.

## 5. Conclusions

A 28-day ESAH treatment reduces the blood pressure levels in 2K1C-ovariectomized rats. These effects are associated with an important antioxidant and antinitrosant action, in addition to a significant saluretic effect. Probably, this response has a direct contribution of the reduction in vascular resistance, possibly mediated by the release of NO and prostaglandins.

## Figures and Tables

**Table 1:** Phytochemical composition of ESAH obtained from *A. hispidum* DC.

Peak	Rt (min)	MS^1^	MS^2^ (key fragments)	Tentative identification
1	2.30	353.08773 [M-H]^−^	191.055, 179.034, 135.045	*neo*-Chlorogenic acid
2	3.22	353.08745 [M-H]^−^	191.055	Chlorogenic acid
3	3.54	353.08773 [M-H]^−^	191.055, 179.034, 173.045, 135.044	*crypto*-Chlorogenic acid
4	5.94	463.08838 [M-H]^−^	300.027	Quercetin-galactoside
5	6.05	463.08804 [M-H]^−^	300.027	Quercetin-glucoside
6	6.43	515.11964 [M-H]^−^	353.087, 335.076, 191.055, 179.034, 173.045, 135.045	3,4-Dicaffeoylquinic acid
7	6.67	515.11970 [M-H]^−^	353.087, 191.055, 179.034,173.045, 135.044	3,5-Dicaffeoylquinic acid
8	7.07	515.11951 [M-H]^−^	353.087, 191.055, 179.034,173.045, 135.044	4,5-Dicaffeoylquinic acid
9	7.28	535.26876 [M+Cl]^−^	337.238	C_26_H_44_O_9_Cl
10	7.54	337.23821 [M-H]^−^	—	C_20_H_33_O_4_
11	8.52	337.23840 [M-H]^−^	—	C_20_H_33_O_4_

**Table 2:** Effects of oral administration of purified aqueous extract obtained from *A. hispidum* DC. (ESAH) on urinary volume and electrolyte excretion, pH, and density on 1st day of treatment.

Group	Urinary volume	El_Na+_	El_k+_	El_Cl−_	El_Ca++_	pH	Density
	(ml/100 g/8 h)	(*µ*Eq/min/100 g)	(*µ*Eq/min/100 g)	(*µ*Eq/min/100 g)	(*µ*Eq/min/100 g)		
Sham	5.64 ± 0.70	0.37 ± 0.06	0.49 ± 0.06	0.46 ± 0.03	0.036 ± 0.0100	7.96 ± 0.15	1016 ± 1.89
C+	4.99 ± 0.27	0.36 ± 0.02	0.44 ± 0.03	0.42 ± 0.02	0.046 ± 0.0058	7.91 ± 0.07	1019 ± 0.66
ESAH (30 mg/kg)	3.81 ± 0.46	1.06 ± 0.07^ab^	0.67 ± 0.06	1.22 ± 0.08^ab^	0.033 ± 0.0068	7.82 ± 0.19	1014 ± 2.23
ESAH (100 mg/kg)	3.96 ± 0.36	0.92 ± 0.19^ab^	0.53 ± 0.07	1.08 ± 0.23^ab^	0.034 ± 0.0041	7.81 ± 0.13	1019 ± 2.10
ESAH (300 mg/kg)	3.44 ± 0.26	0.80 ± 0.12^ab^	0.49 ± 0.07	1.00 ± 0.08^ab^	0.036 ± 0.0068	7.43 ± 0.14	1018 ± 1.76
ENAL	4.72 ± 0.59	0.27 ± 0.05	0.55 ± 0.03	0.37 ± 0.06	0.041 ± 0.0019	7.96 ± 0.15	1017 ± 0.97
HCTZ	7.61 ± 0.28^a^	0.99 ± 0.24^ab^	0.75 ± 0.17	1.18 ± 0.30^ab^	0.030 ± 0.0069	7.80 ± 0.13	1015 ± 1.17

Values are expressed as mean ± SEM of 6-7 rats in each group in comparison with the positive control (C+; ^a^*p* < 0.05) or Sham-operated group (^b^*p* < 0.05) using one-way ANOVA followed by Dunnett's test. El: excreted load; HCTZ: hydrochlorothiazide; ENAL: enalapril.

**Table 3:** Effects of oral administration of purified aqueous extract obtained from *A. hispidum* DC. (ESAH) on urinary volume and electrolyte excretion, pH, and density on 7th day of treatment.

Group	Urinary volume	El_Na+_	El_k+_	El_Cl−_	El_Ca++_	pH	Density
	(ml/100 g/8 h)	(*µ*Eq/min/100 g)	(*µ*Eq/min/100 g)	(*µ*Eq/min/100 g)	(*µ*Eq/min/100 g)		
Sham	9.16 ± 1.09	0.43 ± 0.04	0.40 ± 0.06	0.47 ± 0.04	0.036 ± 0.0064	7.96 ± 0.14	1009 ± 0.70
C+	5.56 ± 0.21	0.49 ± 0.06	0.64 ± 0.08	0.59 ± 0.07	0.050 ± 0.0034	8.06 ± 0.07	1011 ± 1.18
ESAH (30 mg/kg)	4.72 ± 0.57	1.05 ± 0.08^ab^	0.44 ± 0.04	1.12 ± 0.07^ab^	0.037 ± 0.0061	7.48 ± 0.16	1014 ± 1.80
ESAH (100 mg/kg)	4.85 ± 0.71	0.98 ± 0.18^ab^	0.73 ± 0.11	1.28 ± 0.25^ab^	0.059 ± 0.0032	7.95 ± 0.18	1011 ± 1.87
ESAH (300 mg/kg)	4.87 ± 0.61	0.83 ± 0.11	0.49 ± 0.08	0.94 ± 0.13^ab^	0.032 ± 0.0089	7.55 ± 0.12	1014 ± 1.82
ENAL	6.03 ± 0.46	0.37 ± 0.03	0.48 ± 0.04	0.53 ± 0.03	0.009 ± 0.0018	8.05 ± 0.14	1009 ± 1.02
HCTZ	6.85 ± 0.47	0.60 ± 0.07	0.72 ± 0.05	0.79 ± 0.08	0.013 ± 0.0048	7.85 ± 0.11	1011 ± 0.84

Values are expressed as mean ± SEM of 6-7 rats in each group in comparison with the positive control (C+; ^a^*p* < 0.05) or Sham-operated group (^b^*p* < 0.05) using one-way ANOVA followed by Dunnett's test. El: excreted load; HCTZ: hydrochlorothiazide; ENAL: enalapril.

**Table 4:** Effects of oral administration of purified aqueous extract obtained from *A. hispidum* DC. (ESAH) on urinary volume and electrolyte excretion, pH, and density on 14th day of treatment.

Group	Urinary volume	El_Na+_	El_k+_	El_Cl−_	El_Ca++_	pH	Density
	(ml/100 g/8 h)	(*µ*Eq/min/100 g)	(*µ*Eq/min/100 g)	(*µ*Eq/min/100 g)	(*µ*Eq/min/100 g)		
Sham	10.36 ± 1.32	0.53 ± 0.06	0.50 ± 0.06	0.60 ± 0.08	0.050 ± 0.0056^b^	7.96 ± 0.16	1014 ± 0.70
C+	7.11 ± 0.62	0.36 ± 0.04	0.41 ± 0.03	0.42 ± 0.05	0.052 ± 0.0049	7.60 ± 0.08	1014 ± 0.86
ESAH (30 mg/kg)	5.66 ± 0.23	0.50 ± 0.04	0.68 ± 0.06	0.60 ± 0.04	0.030 ± 0.0052	8.07 ± 0.05	1017 ± 0.89
ESAH (100 mg/kg)	4.78 ± 0.64	0.88 ± 0.20	0.50 ± 0.06	1.01 ± 0.24	0.036 ± 0.0083	7.61 ± 0.20	1018 ± 2.12
ESAH (300 mg/kg)	5.76 ± 0.57	1.16 ± 0.03^ab^	0.74 ± 0.07	1.33 ± 0.04^ab^	0.036 ± 0.0069	7.95 ± 0.08	1019 ± 0.88
ENAL	6.01 ± 0.73	0.53 ± 0.08	0.56 ± 0.03	0.71 ± 0.08	0.026 ± 0.0046	8.04 ± 0.03	1015 ± 1.02
HCTZ	6.55 ± 0.33	0.74 ± 0.11	0.65 ± 0.10	0.92 ± 0.15	0.055 ± 0.0232	8.06 ± 0.03	1014 ± 1.15

Values are expressed as mean ± SEM of 6-7 rats in each group in comparison with the positive control (C+; ^a^*p* < 0.05) or Sham-operated group (^b^*p* < 0.05) using one-way ANOVA followed by Dunnett's test. El: excreted load; HCTZ: hydrochlorothiazide; ENAL: enalapril.

**Table 5:** Effects of oral administration of purified aqueous extract obtained from *A. hispidum* DC. (ESAH) on urinary volume and electrolyte excretion, pH, and density on 21st day of treatment.

Group	Urinary volume	El_Na+_	El_k+_	El_Cl−_	El_Ca++_	pH	Density
	(ml/100 g/8 h)	(*µ*Eq/min/100 g)	(*µ*Eq/min/100 g)	(*µ*Eq/min/100 g)	(*µ*Eq/min/100 g)		
Sham	8.98 ± 0.93	0.46 ± 0.06	0.48 ± 0.05	0.52 ± 0.07	0.041 ± 0.0070	7.87 ± 0.12	1015 ± 2.72
C+	7.16 ± 0.42	0.59 ± 0.07	0.76 ± 0.11	0.74 ± 0.10	0.068 ± 0.0078	8.10 ± 0.08	1014 ± 1.11
ESAH (30 mg/kg)	6.73 ± 0.72	0.73 ± 0.12	0.72 ± 0.18	0.99 ± 0.17	0.043 ± 0.0054	8.25 ± 0.17	1014 ± 2.01
ESAH (100 mg/kg)	4.82 ± 0.33	0.77 ± 0.16	0.75 ± 0.20	0.75 ± 0.19	0.046 ± 0.0054	8.30 ± 0.09	1018 ± 0.85
ESAH (300 mg/kg)	5.20 ± 1.23	1.21 ± 0.20^ab^	0.74 ± 0.06	1.47 ± 0.21^ab^	0.039 ± 0.0033	7.85 ± 0.07	1019 ± 1.70
ENAL	5.30 ± 0.25	0.30 ± 0.06	0.51 ± 0.05	0.47 ± 0.06	0.038 ± 0.0091	8.02 ± 0.06	1017 ± 1.72
HCTZ	6.22 ± 0.48	0.45 ± 0.06	0.41 ± 0.02	0.55 ± 0.05	0.019 ± 0.0058	7.88 ± 0.06	1014 ± 0.95

Values are expressed as mean ± SEM of 6-7 rats in each group in comparison with the positive control (C+; ^a^*p* < 0.05) or Sham-operated group (^b^*p* < 0.05) using one-way ANOVA followed by Dunnett's test. El: excreted load; HCTZ: hydrochlorothiazide; ENAL: enalapril.

**Table 6:** Effects of oral administration of purified aqueous extract obtained from *A. hispidum* DC. (ESAH) on urinary volume and electrolyte excretion, pH, and density on 28th day of treatment.

Group	Urinary volume	El_Na+_	El_k+_	El_Cl−_	El_Ca++_	pH	Density
	(ml/100 g/8 h)	(*µ*Eq/min/100 g)	(*µ*Eq/min/100 g)	(*µ*Eq/min/100 g)	(*µ*Eq/min/100 g)		
Sham	7.84 ± 0.55	0.44 ± 0.03	0.49 ± 0.03	0.53 ± 0.04	0.034 ± 0.0020	8.02 ± 0.04	1016 ± 1.36
C+	5.92 ± 0.32	0.28 ± 0.02	0.37 ± 0.07	0.34 ± 0.04	0.027 ± 0.0047	8.00 ± 0.20	1018 ± 0.92
ESAH (30 mg/kg)	4.85 ± 0.41	0.52 ± 0.04	0.66 ± 0.07	0.58 ± 0.04	0.037 ± 0.0135	8.41 ± 0.05	1015 ± 1.08
ESAH (100 mg/kg)	4.68 ± 0.63	0.29 ± 0.02	0.43 ± 0.04	0.40 ± 0.03	0.027 ± 0.0069	8.34 ± 0.07	1017 ± 2.04
ESAH (300 mg/kg)	3.94 ± 0.49	0.82 ± 0.09^ab^	0.77 ± 0.05	1.14 ± 0.12^ab^	0.042 ± 0.0060	8.01 ± 0.08	1019 ± 1.08
ENAL	5.13 ± 0.61	0.72 ± 0.13	0.67 ± 0.10	0.88 ± 0.13	0.046 ± 0.0061	8.31 ± 0.05	1014 ± 1.64
HCTZ	6.89 ± 0.45	0.80 ± 0.09^ab^	0.62 ± 0.07	0.96 ± 0.11	0.032 ± 0.0133	8.04 ± 0.05	1017 ± 0.62

Values are expressed as mean ± SEM of 6-7 rats in each group in comparison with the positive control (C+; ^a^*p* < 0.05) or Sham-operated group (^b^*p* < 0.05) using one-way ANOVA followed by Dunnett's test. El: excreted load; HCTZ: hydrochlorothiazide; ENAL: enalapril.

**Table 7:** Effects of oral administration of purified aqueous extract obtained from *A. hispidum* DC. (ESAH) on serum Na^+^, K^+^, urea, and creatinine on 28th day of treatment.

Group	Na^+^ (mmol/L)	K^+^ (mmol/L)	Urea (mg/dL)	Creatinine (mg/dL)
Sham	136.0 ± 0.35	4.81 ± 0.21	53.37 ± 1.37	0.37 ± 0.01
C+	119.1 ± 3.83	4.47 ± 0.20	55.07 ± 4.73	0.36 ± 0.02
ESAH (30 mg/kg)	131.7 ± 1.70	6.16 ± 0.50	63.68 ± 3.58	0.44 ± 0.03
ESAH (100 mg/kg)	131.2 ± 5.54	4.95 ± 0.47	54.43 ± 2.90	0.34 ± 0.01
ESAH (300 mg/kg)	119.4 ± 8.89	4.74 ± 0.41	49.40 ± 2.08	0.36 ± 0.01
ENAL	134.4 ± 1.36	5.21 ± 0.10	63.90 ± 1.35	0.40 ± 0.01
HCTZ	136.5 ± 0.63	4.85 ± 0.21	62.15 ± 2.64	0.35 ± 0.03

Values are expressed as mean ± SEM of 6-7 rats in each group in comparison with the positive control (C+) or Sham-operated group using one-way ANOVA followed by Dunnett's test. HCTZ: hydrochlorothiazide; ENAL: enalapril.
